# A Gas Chromatography Mass Spectrometry-Based Method for the Quantification of Short Chain Fatty Acids

**DOI:** 10.3390/metabo12020170

**Published:** 2022-02-11

**Authors:** Julia K. Rohde, Marceline M. Fuh, Ioannis Evangelakos, Mira J. Pauly, Nicola Schaltenberg, Francesco Siracusa, Nicola Gagliani, Klaus Tödter, Joerg Heeren, Anna Worthmann

**Affiliations:** 1 Department of Biochemistry and Molecular Cell Biology, University Medical Center Hamburg-Eppendorf, 20246 Hamburg, Germany; jul.rohde@uke.de (J.K.R.); m.fuh@uke.de (M.M.F.); i.evangelakos@uke.de (I.E.); mirapauly@web.de (M.J.P.); nicolaschaltenb@aol.com (N.S.); klaustoedter@arcor.de (K.T.); heeren@uke.de (J.H.); 2 I. Department of Medicine and Department of General, Visceral and Thoracic Surgery, University Medical Center Hamburg-Eppendorf, 20246 Hamburg, Germany; f.siracusa@uke.de (F.S.); n.gagliani@uke.de (N.G.); 3 Hamburg Center for Translational Immunology (HCTI), University Medical Center Hamburg-Eppendorf, 20246 Hamburg, Germany

**Keywords:** SCFA, GC-MS, gut bacteria, fermentation, feces

## Abstract

Short Chain Fatty Acids (SCFAs) are produced by the gut microbiota and are present in varying concentrations in the intestinal lumen, in feces but also in the circulatory system. By interacting with different cell types in the body, they have a great impact on host metabolism and their exact quantification is indispensable. Here, we present a derivatization-free method for the gas chromatography mass spectrometry (GC-MS) based quantification of SCFAs in plasma, feces, cecum, liver and adipose tissue. SCFAs were extracted using ethanol and concentrated by alkaline vacuum centrifugation. To allow volatility for separation by GC, samples were acidified with succinic acid. Analytes were detected in selected ion monitoring (SIM) mode and quantified using deuterated internal standards and external calibration curves. Method validation rendered excellent linearity (R^2^ > 0.99 for most analytes), good recovery rates (95–117%), and good reproducibility (RSD: 1–4.5%). Matrix effects were ruled out in plasma, feces, cecum, liver and fat tissues where most abundant SCFAs were detected and accurately quantified. Finally, applicability of the method was assessed using samples derived from conventionally raised versus germ-free mice or mice treated with antibiotics. Altogether, a reliable, fast, derivatization-free GC-MS method for the quantification of SCFAs in different biological matrices was developed allowing for the study of the (patho)physiological role of SCFAs in metabolic health.

## 1. Introduction

Short chain fatty acids (SCFAs) are fatty acids with six or fewer carbon atoms and are produced by microbial fermentation of indigestible carbohydrates in the colon [1]. The predominant SCFAs are acetic acid, propionic acid and butyric acid. The vast majority of SCFAs are absorbed by colonocytes and only small proportions of SCFAs are excreted with the feces. Besides their role as energy substrates for colonocytes and hepatocytes, SCFAs are ligands of G-protein coupled receptors (GPRs) and they have recently been shown to be implicated in the regulation of appetite [2,3], development of obesity [4,5] and fatty liver [6] as well as insulin sensitivity and energy expenditure [7]. SCFAs can act systemically or in metabolically active tissues, as acetate is able to suppress insulin signaling in adipose tissues via GPR43 [5]. SCFA production can be enhanced by dietary interventions, and leveraging the diet–gut–microbiota–SCFA axis for therapeutic purposes is an active area of research. Accordingly, fast, convenient, accurate and reliable analytical techniques for the quantification of SCFAs are required.

The high volatility and hydrophilic nature of SCFAs together with their appearance in complex biological matrices make SCFAs a demanding analytical target. Gas chromatography mass spectrometry-based techniques have emerged as the method of choice for the quantification of SCFAs. As GC-MS requires suitable volatile compounds, fatty acids are commonly derivatized into their methyl ester or trimethylsilyl ester derivatives. However, due to the low intrinsic boiling point of SCFA, which is similar to that of commonly used derivatization agents, signal overlap might occur and therefore the derivatization agent has to be chosen carefully. Additionally, many derivatization agents are moisture-sensitive and thus not suitable for the aqueous matrices containing SCFAs. These shortcomings have been circumvented by using isobutyl chloroformate [8] and pentafluorbenzylbromid (PFBBr) [9]. Furthermore, recent derivatization-free approaches using solid-phase microextraction (SPME) for the sample cleanup have been developed [10,11]. Another alternative and derivatization-free approach for the GC-based analysis of SCFAs involves the direct analysis of acidified water/extracts using HCl [12] or phosphoric acid [13]. Yet these methods might render low recoveries and bear the risk of impurities and GC column contaminations. Here we present an improved method, where we combine a rapid and convenient sample cleanup involving an ethanolic extraction and succinic acid-mediated acidification with a state-of-the-art GC-MS-based detection for the accurate and reliable quantification of SCFAs. Contrary to HCl and phosphoric acid, succinic acid is a mild acid and easier to handle. Of note, our method is validated not only for feces and plasma samples as previous methods, but also for metabolically active tissues such as liver and adipose tissue, which are known be affected by SCFA signaling via GPRs.

## 2. Results and Discussion

### 2.1. GC-MS Method

**Figure 1:**
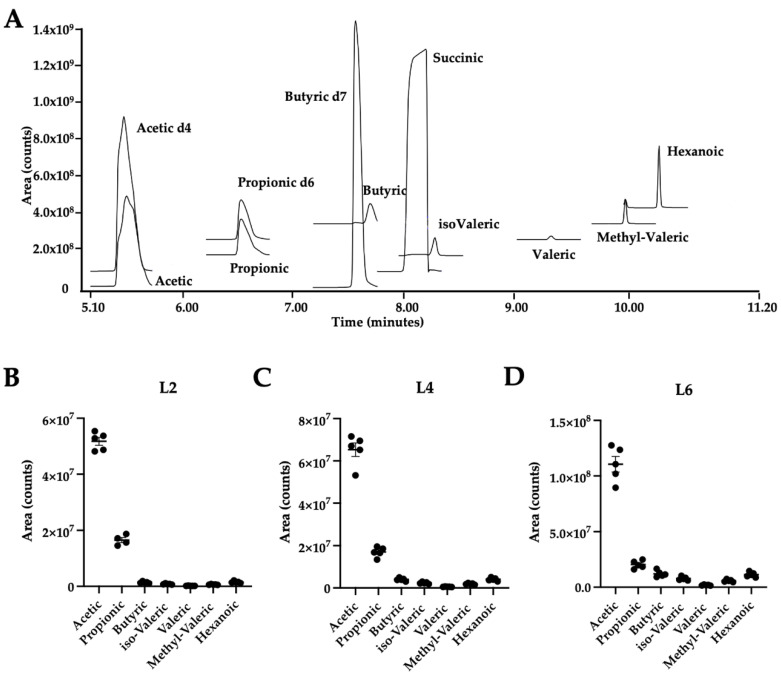
(**A**) Exemplary chromatogram of a mixed standard solution (**B**–**D**) Repeatability of each analyte was assessed at 3 different concentration ranges (level 2 = L2, level 4 = L4, level 6 = L6). L2 (**B**), L4 (**C**) and L6 (**D**) were injected 5 times on different days and areas are shown.

The exact and reliable quantification of SCFAs is essential for understanding their role and mode of action in the prevention and progression of several diseases. Consequently, we aimed to set up a method for the quantification of SCFAs in various biological matrices, especially in those tissues where SCFAs might actually modify metabolic responses such as the liver and the adipose tissues [4,7]. Additionally, we aimed to establish a fast and convenient sample cleanup which does not depend on derivatization. For this purpose, a Nukol^TM^ column was employed which has an acidic character due to its acid-modified poly (ethylene glycol) phase and is thus well suited for the separation of volatile acidic compounds. Similar free fatty acid phase columns have been applied successfully before [12,14]. The use of the Nukol^TM^ column was combined with the succinate-mediated acidification of our ethanolic standard solutions. Of note, succinic acid is environmentally friendly and easier to handle than the previously used HCl and phosphoric acid. Firstly, the GC method was optimized to yield a good separation of all the SCFA compounds, as they all have similar target ion masses (Table 1). In addition, a goal was set to clearly separate the acetic acid peak from the peak of injection. As shown in Figure 1A, we achieved a clear separation of all analytes. All analytes were identified using the NIST database, to which the obtained spectra were compared. In addition to the analyte peaks, a peak of succinic acid was also detected, which was the compound used to improve volatility of the analytes (Figure 1A). Target (TI) and confirmative ion (CI) *m/z* for each analyte and their respective internal standards were chosen based on signal intensity. Quantification was performed from data acquired in timed SIM mode using the retention times (RT) and TI/CI *m/z* given in Table 1 and Table A1. 

### 2.2. Calibration Curve, Limit of Quantification, Carry Over, Accuracy and Precision

In order to assess linearity of the method, an 8-point calibration was run and back-calculation of the calibrators was performed (Table A2). In line with the EMA criteria [15], 80% of the back-calculated concentrations differed only 15% from the nominal values and thus linearity was given with exception of butyric acid, methyl-valeric acid and hexanoic acid, where only seven calibration points were included. The linearity ranges, equations of the regression curves, correlation coefficients (R^2^), limits of detection (LOD) and limits of quantitation (LOQ) are shown in Table 1. All major SCFAs, including acetic acid, propionic acid and butyric acid, displayed a (R^2^) ≥ 0.98 in the given concentration ranges, which is comparable to those of the other methods described before [12,13,16]. Carry-over was assessed for each analyte and internal standards (Table 1 and Table A1). The highest carry-over was observed for butyric acid (7.8% of LOQ) and hexanoic acid (7.3% of LOQ) but all values were below 20% of LOQ (Table 1), as required by the EMA guidelines [15]. The next goal was to assess the repeatability of the method and calculate the accuracy and the precision for each analyte at different levels of the 8-point calibration curves to cover different concentration ranges. In line with the EMA criteria [15], the obtained values for accuracy (given as relative error percentages RE %) were below 20% for all analytes throughout the concentration ranges, except from at the LOQ (see Table A2). Additionally, at the second lowest concentration level (L2), the accuracy of the butyric acid, methyl-valeric acid and hexanoic acid were slightly above 20% (butyric acid, methyl-valeric acid) and at 48% for hexanoic acid. As stated before, for these analytes only seven calibration points were used and the linear range was adjusted accordingly. Regarding accuracy, as shown in Figure 1B–D, the areas of individual measurements are clustered close to the mean. With the exception of valeric acid, for most analytes and most levels, the relative standard deviation (RSD) was in the range of 1% to 4.5% (Table A2) and thus below the required 15% according to EMA guidelines [15]. Of note, in the middle to higher range levels (L6 and L8), RSD was even between 1% and 3% (Table A2). Interestingly, contrary to other methods [12,13], our method was especially reliable for acetic acid throughout all concentration ranges (RSD: 1–2.6%). In addition to the accuracy and precision values determined using the calibration standards, we also assessed accuracy and precision in the quality control (QC) samples, plasma and caecum samples spiked with known and physiologic concentrations of the analytes. As given in Table A3, precision in the QC samples did not exceed 15% for all analytes and was thus in the line with the EMA requirements. However, with exception of acetate, accuracy ranged from 23–37% in caecum QC samples but did not exceed 20% in plasma samples. Overall, these data indicate that our method was accurate and precise. 

### 2.3. Sample Extraction and Recovery

**Figure 2:**
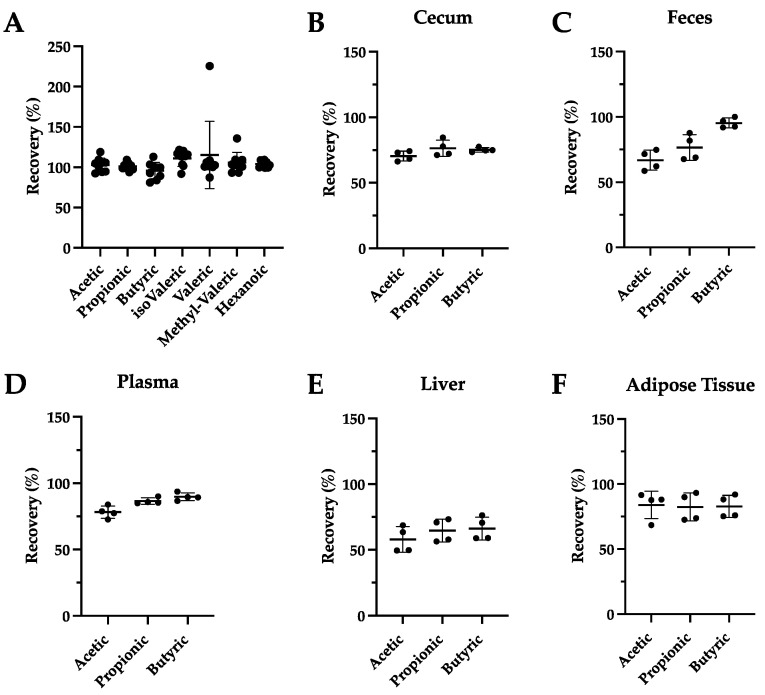
(**A**) Recovery percentages of a standard mixture using acidification with succinic acid. Recovery percentages of deuterated acetic acid, propionic acid and butyric acid in cecum (**B**), feces (**C**), plasma (**D**), liver (**E**) and adipose tissue (**F**).

**Figure A1:**
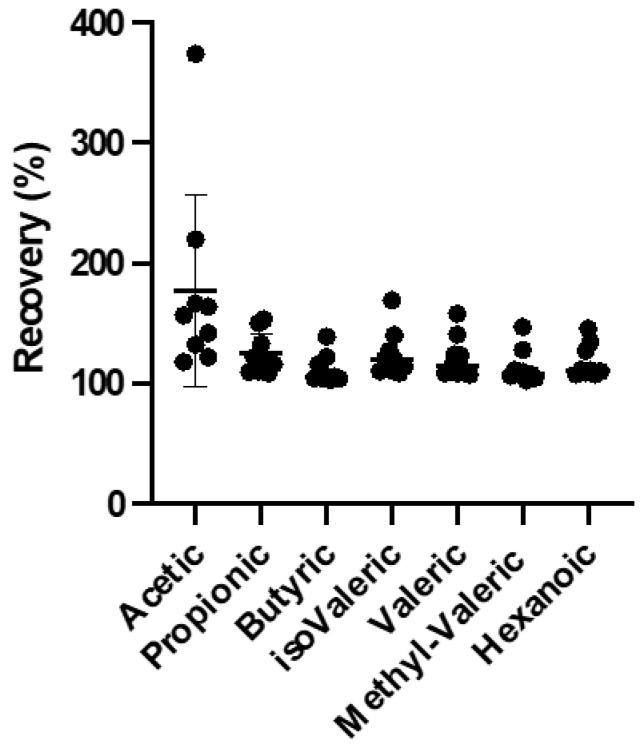
Recovery percentages of a standard mixture of SCFA using acidification with phosphoric acid.

One of our aims was to establish an easy and derivatization-free sample clean up with as few column contaminations as possible. For this purpose, acidified water samples were not used [17] as they are prone to contaminating the GC column, but rather a combination of an ethanolic sample extraction with acidification was used. As it has been proposed that a low pH promotes SCFA solubility and recoveries [12], acidification of samples is performed before their extraction in organic solvents [1,13]. Nevertheless, in our method, the ethanolic extraction was first carried out before acidifying the samples right before the GC analysis. Due to its easy handling, succinic acid was tried for acidification and compared to the acidification achieved by phosphoric acid as described before [13]. The amount of sample used for extraction (30 mg) was quite low compared to other methods, using 50–100 mg or even 1 g [8,9,12,13]. In order to test the extraction yield of the different acidification steps, recovery analyses were performed. For this purpose, a standard mix solution with known concentrations was measured directly, or after extraction with either succinic or phosphoric acid according to our extraction protocol. As indicated in Figure 2A, recovery was in the range of 95–117% for all analytes using succinic acid for acidification. However, as shown in Figure A1 and summarized in Table A4, recoveries for phosphoric acid ranged between 111 and 177%. As recovery rates were better using succinic acid, we decided to use succinic acid for acidification. Next, recovery was determined in a matrix containing samples by measuring concentrations of deuterated internal standards in indicated tissue samples which were either spiked with IS mix solution before or after extraction. Of note here, different recoveries amongst the tissues analyzed were detected but also amongst the analytes in the tissues themselves (Figure 2B–F). While in the liver, recovery rates for all IS ranged between 58% and 66% (Figure 2E), recoveries of 75 to 100% were calculated for most of the deuterated standards in all other tissues except from the deuterated acetic acid in fecal samples (67%) (Figure 2B–D,F). As expected, the overall recovery was best (acetic 78%, propionic 87% and butyric 90%) in the plasma samples (Figure 2D) but the highest recovery was achieved for butyric acid in the feces samples (95%) (Figure 2C). In all tissues, recovery of acetic acid was most challenging (Figure 2B–F), which is in line with previous reports [13,16] but still above 75%, except in the liver and the feces. Overall, recoveries were similar or even better than in other reports [8,13]. Of note, while others determine recovery mostly in feces samples [12,13,16], recovery studies were performed in a variety of other tissues in this study. In conclusion, the extraction method presented here is well suited for the reliable and fast extraction of SCFAs from various tissues.

### 2.4. Matrix Effects 

**Figure 3:**
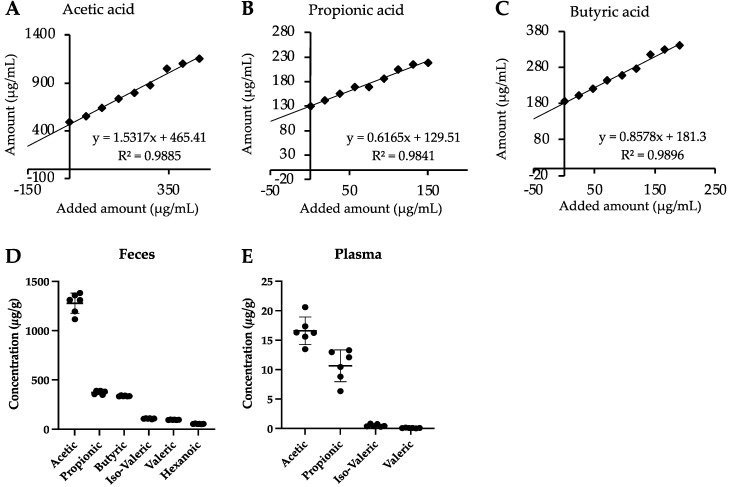
Standard addition method applied to acetic acid (**A**), propionic acid (**B**) and butyric acid (**C**) in cecum samples. The same feces (**D**) and plasma (**E**) sample were measured 6 times on different days to assess reproducibility.

SCFAs are typically present in intestinal tissues as well as in the gastrointestinal lumen, which are both complex biological matrices. To rule out potential matrix effects on detection signals, a standard addition for every tissue analyzed was performed. As depicted in Figure 3A–C for cecum samples, signal areas of acetic acid, propionic acid and butyric acid were a linear function of the added concentrations. This was also true for other tissues (see R^2^ in Table A4). Furthermore, the SCFA concentrations in the fecal samples calculated by regression analysis from the standard addition curves were similar to the concentrations calculated by external calibration. However, for plasma and for caecum samples the concentrations were more divergent (Table A5). We thus assessed the matrix effects in accordance with the EMA guidelines [15] and calculated the coefficient of variation (CV) for each analyte in each matrix at two concentration levels. As given in Table A6, while at the lower concentration level the CV of acetic acid (in feces and cecum), butyric acid (in caecum) and valeric acid (feces) was only <35% and all other CV-values and thus more than 90% were <15% and in agreement with the EMA criteria. These results indicate that the method presented here is robust, applicable and reliable not only in feces, cecum and plasma samples as in other methods [8,9,12,13,16], but also in tissue which have been described to be directly affected by SCFAs, such as the liver and adipose tissue [18]. Next, to assess reproducibility in matrix-containing samples, we analyzed feces (Figure 3D) and plasma (Figure 3E) samples on three different days. In between the measurements, the samples were stored at −20 °C. As given in Figure 3D, signal areas in fecal samples obtained on different days clustered closely together for all analytes, with mild deviation in acetic acid. In plasma samples, we detected only acetic acid, propionic acid, valeric acid and iso-valeric acid. In addition, samples varied only mildly around the mean. As summarized in Table A7, RSD values for each analyte except from acetate were in the range between 1.4% and 4.7% in the feces samples, while variation was higher in the plasma. Altogether, the reproducibility in the matrix-containing samples was satisfying, further strengthening the reliability of the method. 

### 2.5. Method Application

**Figure 4:**
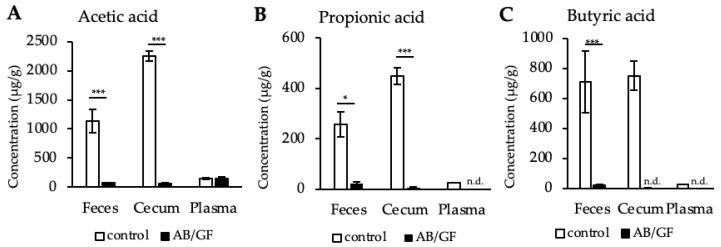
Acetic acid (**A**), propionic acid (**B**) and butyric acid (**C**) levels in feces, cecum and plasma samples of control and either antibiotics (AB) –treated (feces) or germ free (GF) (cecum and plasma) mice (*n* = 5–7;). n.d. = non detectable, Data are shown as mean values ± SD, * indicate significant differences between groups (*: *p* < 0.05, ***: *p* < 0.001) determined by student’s *t*-test.

The link between diet, gut microbes and their metabolites, such as SCFA, is of particular interest for the understanding and prevention of several diseases including obesity and associated inflammatory disorders such as fatty liver disease [19,20]. Mice lacking gut microbes either due to breeding in germ free (GF) facilities or due to their eradication by antibiotics (AB) display substantially affected SCFA levels. In particular, both germ free mice and AB-treated mice have strongly reduced SCFA levels [21,22]. As a proof of principle, we applied the GC-MS based method presented here for the quantification of SCFA on various murine tissue samples from normal control mice, mice treated with AB or GF mice. Figure 4 shows acetic acid (Figure 4A), propionic acid (Figure 4B) and butyric acid (Figure 4C) levels in the feces, cecum and plasma samples of control and either AB (feces samples) or GF (cecum and plasma samples) mice. As expected, levels of all major SCFA were significantly reduced in AB/GF mice compared to control mice. In line with a previous report [23], butyric acid was particularly dramatically affected by microbiota depletion. In particular, in cecum and plasma, butyric acid levels were below the limit of quantification (Figure 4C). In summary, we demonstrate that the method presented here is applicable to real biological samples and may help to address relevant biological questions in the future. 

## 3. Materials and Methods

### 3.1. Chemicals

Ethanol was purchased from Merck (Darmstadt, Germany). Phosphoric acid, NaOH, succinic acid and the volatile fatty acid standards were purchased from Sigma (St. Louis, MO, USA). Helium was used as carrier gas for GC-MS (99.999% purity, SOL, Krefeld, Germany).

### 3.2. Standard Solutions and Calibration

Standard solutions of individual SCFA and deuterated SCFA (d-SCFA) were prepared by diluting the respective compound in ethanol. An internal standard (IS) mix solution was prepared with a final concentration of 4000 mg/L of each individual d-SCFA. For every calibration point, the IS mix was used in a final concentration of 500 mg/L for each d-SCFA. The d4-acetic acid was used for quantification of acetic acid, d6-propionic acid was used for quantification of propionic acid and d7-butyric acid was used for quantification of all other SCFA. Right before analysis, each calibration point was diluted 1:6 with 0.6 M succinic acid for acidification.

### 3.3. Sample Preparation

30 mg of tissue or 30 µL of plasma were extracted in 293.75 μL ethanol, and 6.25 µL of deuterated IS mix (c = 4000 mg/L) was added. The samples were homogenized either by vortexing (plasma) or using a Tissue Lyzer (Qiagen) (feces, cecum, liver, fat tissue), and were then centrifuged (10 min, 13,000 *g*). The supernatant was transferred to a fresh tube and 5 μL of 0.8 M NaOH were added before solvents were evaporated using a vacuum centrifuge. The residual salts were redissolved in 50 μL EtOH and acidified with 10 μL 0.6 M succinic acid or 10 µL of 85% phosphoric acid right before the analysis.

### 3.4. GC. MS Analysis

GC-MS analysis was performed by a TRACE 1310 gas chromatograph/ISQ 7000 mass selective detector (ThermoFisher Scientific, Dreieich, Germany) equipped with a Nukol Fused Silica Capillary Column (15 m × 0.32 mm × 0.25 μm film thickness) (Supelco/Sigma Aldrich, St. Louis, MO, USA). The injector, GC-MS transfer line and ion source temperature were set to 200, 200 and 250 °C, respectively. The flow rate of helium carrier gas initially started at 2.5 mL/min, was kept there for 6.2 min and then ramped to 5 mL/min at a rate of 1 mL/min where it was kept for 5.1 min. 1 μL of sample was introduced by splitless injection. The initial column temperature was set to 55 °C and held for 1 min, followed by a ramp up to 105 °C at a rate of 8 °C/min where it was held for 2 min. Finally, the column temperature was increased to 190 °C at a rate of 30 °C/min and kept at this temperature for 1 min. The ionization was carried out in the electron impact (EI) mode at 70 eV. Initially, the MS data were acquired in full scan mode from *m/z* 40–130 with a scan time of 0.2 s. The identification of compounds was achieved by comparing the obtained MS spectra to the NIST database and confirmed by comparison to the retention times of pure standards. The analytes were quantified in the timed selected ion monitoring (SIM) mode using the target ion and verified by confirmative ions. The *m/z* of TI/CIs (*m/z*) are shown in Table 1 and Table A1. Instrument was operated, data were acquired and analyzed using Chromeleon software. Contents of SCFA were calculated using external calibration curves.

### 3.5. Linearity

Linearity of the method was determined by analysis of 8-point calibration curves (*n* = 5) for SCFA standard solutions and back-calculation of the calibrators was performed at every concentration level. Linearity ranges, the regression equation and the coefficient of determination (R^2^) are shown in Table 1.

### 3.6. Limit of Dectection (LOD) and Quantification (LOQ)

LOQ was set as the lowest standard of the 8-point calibration curve, meeting the following conditions: The analyte signal area was at least 20 times higher than the signal of a blank and the analyte peak was identifiable, discrete and reproducible. LOD was estimated by the cut-off approach: replicate measurements near the LOD were performed and the concentration at which the signal of the analyte was 3 times bigger than the signal of the blank was set at LOD [24]. LOD and LOQ are given in Table 1.

### 3.7. Carry Over

Carry-over was assessed by injecting blank samples (*n* = 5) after the highest calibration standard. Carry-over is given as ratio of blank area to area of LOQ in percentage. 

### 3.8. Recovery Assay

Recovery was assessed in two different ways. First, a level of the 8-point calibration curve was either measured directly or after undergoing the extraction procedure as described above. Second, tissue samples were either spiked with IS mix solution before or after the extraction procedure and then measured. For both ways, recovery was calculated as follows: Recovery, % = $\frac{\;concentration{\;}_{\left(with\;extraction/spike\;before\right)}}{concentration_{\left(without\;extraction\;/spike\;after\right)\;}}$.

### 3.9. Standard-Addition

For standard addition, tissues were extracted as described above and initial concentration was assessed. An addition solution containing all analytes in known concentrations was prepared. Differing amounts of addition solution were added to an equal volume of the extracts and all mixes were then brought to the same volume by dilution. Measured concentrations were plotted against the concentration of the addition solution and linear regression was performed. The initial concentration was then recalculated by means of linear regression.

### 3.10. Effects of Matrix Effects

Matrix effects were assessed according to the EMA guidelines [15] at a low (low) and at a high level of concentration (high). Exact concentrations (in mg/L) for each fatty acid were the following (low/high): acetic acid: 25/500, propionic acid 12.5/250, butyric acid 25/500, isovaleric acid: 5/100, valeric acid: 1.25/25, methyl-valeric acid: 10/200, hexanoic acid: 12.5/200. Briefly, for every matrix, an internal standard normalized matrix factor was calculated by dividing the peak area in the presence of the matrix by the peak area in the absence of a matrix for 6 different lots of matrices each. The coefficient of variation (CV) was then calculated for each analyte in each matrix. 

### 3.11. Reproducibilty

The intra- and inter-day repeatability were assessed using either 3/4 different levels from the 8-point calibration curve or using feces and liver extracts. All samples were measured 5 (levels) or 3 (tissues) times. In between, the samples were kept at −20 °C. The accuracy (for the 3/4 different levels from the 8-point calibration curve) was reported as relative error percentages (RE%). The precision was expressed with the relative standard deviation (RSD, %) for corresponding peak areas according to EMA guidelines.

### 3.12. Experimental Animals

For mouse experiments, 5–7 mice per group were used. All animal experiments were conducted in accordance with FELASA guidelines and approved by the Animal Welfare Officers of the University Medical Center Hamburg-Eppendorf (UKE) as well as the Behörde für Gesundheit und Verbraucherschutz Hamburg (animal protocol 15/96, approved 8 October 2015). Mice were housed at the animal facility at 22 °C with a day and night cycle of 12 h with ad libitum access to food and water. Antibiotic treatment was performed by adding bacitracin, neomycin and streptomycin (1 g/L each) to the drinking water. Fresh fecal samples were collected directly from the cages. For the organ and blood harvest, mice were anesthetized with a lethal dose of ketamine and xylazine. Cardiac blood was drawn with syringes containing 5 μL 0.5 M EDTA. Animals were perfused with PBS containing 10 U/mL heparin, then the organs were taken and immediately stored at −80 °C for further analysis.

### 3.13. Statistical Analysis

Data are expressed as mean ± S.D. Statistical analysis was performed using GraphPad Prism 9.0. Two-tailed, independent Student’s *t* test was assessed to compare differences between groups. Differences were considered as significant at a probability level (*p*) of 0.05. 

## 4. Conclusions

In conclusion, the method presented herein for the analysis of SCFAs combines a convenient sample cleanup with the reproducible quantification of SCFAs. Moreover, the advantages of this method are the low amount of required tissue samples and the applicability in a variety of tissues which might help to complement the complex picture of SCFA-mediated systemic (patho)physiological responses.

## Figures and Tables

**Table 1:** Mass/charge (*m/z*) of target ion (TI), confirmative ion 1–2 (CI1, CI2), retention time (RT), regression linear equation, correlation coefficient (R^2^), limit of detection (LOD), limit of quantification (LOQ), linearity ranges and carry over of the SCFAs.

Analyte	*m/z* (TI, CI1, CI2)	RT (min)	Regression Linear Equation	R^2^	LOD (µg/mL)	LOQ (µg/mL)	Linearity Ranges (µg/mL)	Carry Over (% LOQ)
Acetic acid	43, 45, 60	5.4	Y = 0.0029x + 0.4198	0.9999	0.5	10	10–800	1.0006
Propionic acid	29, 45, 74	6.5	Y = 0.0017x + 0.9075	0.9974	1	5	5–400	0.2535
Butyric acid	41, 60, 73	7.5	Y = 0.0006x + 0.0083	0.9965	0.13	10	10–500	7.8136
Iso-Valeric acid	43, 60, 87	8.3	Y = 0.0019x + 0.0004	0.9998	0.2	2	2–160	6.3859
Valeric acid	41, 60, 73	9.4	Y = 0.002x − 0.0005	0.9998	0.01	0.5	0.5–40	5.5461
4-Methylvaleric Acid	43, 57, 74	10.1	Y = 0.0006x + 0.0030	0.9981	0.05	4	4–200	5.9567
Hexanoic acid	60, 73, 87	10.4	Y = 0.0009x + 0.0130	0.9906	0.1	5	5–250	7.2714

## Data Availability

The raw data of this article will be made available by the authors upon request. The data are not publicly available due to restrictions.

## Appendix A

**Table A1:** Retention time (RT), mass/charge (*m/z*), analytes used for deuterated internal standards and carry over (% limit of quantification LOQ).

Analyte	*m/z* (TI, CI1, CI2)	RT	Used for	Carry Over (% LOQ)
Acetic acid d4	46. 63	5.4	Acetic acid	0.15
Propionic acid d6	30. 46; 79	6.5	Propionic acid	0.12
Butyric acid d7	63, 77	7.5	Butyric acid, Isovaleric acid Valeric acid 4-Methylvaleric acid Hexanoic acid	0.13

**Table A2:** Repeatability assessment of the method for each analyte using precision given as relative standard deviation RSD (%), accuracy given as relative error (RE) (%) and back-calculated concentration (BCC in µg/mL) of individual analytes at every given concentration (C in µg/mL).

**Analyte**	**L1**				**L2**				**L3**			
	**C** **(µg/mL)**	**BCC** **(µg/mL)**	**RSD** **(%)**	**Accuracy** **RE (%)**	**C** **(µg/mL)**	**BCC** **(µg/mL)**	**RSD** **(%)**	**Accuracy RE (%)**	**C** **(µg/mL)**	**BCC** **(µg/mL)**	**RSD** **(%)**	**Accuracy RE (%)**
Acetic acid	10	9.94	2.49	0.58	25	26.27	2.65	5.07	50	50.45	2.38	0.89
Propionic acid	5	7.41	7.41	48.28	12.5	13.62	6.14	8.98	25	24.85	3.48	0.59
Butyric acid	10	0.65	5.95	93.48	25	19.73	3.29	21.07	50	47.86	4.05	4.29
Iso-Valeric acid	2	1.73	1.77	13.40	5	4.87	3.87	2.56	10	9.57	2.27	4.32
Valeric acid	0.5	0.75	14.92	50.56	1.25	1.40	11.03	11.66	2.5	2.55	10.45	1.90
4-Methylvaleric Acid	4	0.34	5.27	91.56	10	7.81	2.85	21.92	10	19.51	3.68	2.43
Hexanoic acid	5	0.00	6.07	0.00	12.5	6.46	6.12	48.35	25	24.09	4.13	3.66
**Analyte**	**L4**				**L5**				**L6**			
	**C** **(µg/mL)**	**BCC** **(µg/mL)**	**RSD** **(%)**	**Accuracy** **RE (%)**	**C** **(µg/mL)**	**BCC** **(µg/mL)**	**RSD** **(%)**	**Accuracy RE (%)**	**C** **(µg/mL)**	**BCC** **(µg/mL)**	**RSD** **(%)**	**Accuracy RE (%)**
Acetic acid	75	75.30	1.75	0.40	100	101.63	0.79	1.63	250	242.84	1.57	2.87
Propionic acid	37.5	40.09	4.36	6.92	50	55.12	3.60	10.23	125	128.61	2.96	2.89
Butyric acid	75	76.85	2.16	2.47	100	109.68	2.79	9.68	250	260.25	2.99	4.10
Iso-Valeric acid	15	14.63	2.19	2.49	20	20.26	0.72	1.28	50	50.41	1.47	0.82
Valeric acid	3.75	3.68	5.66	1.81	5	4.90	0.85	2.04	12.5	12.02	1.36	3.85
4-Methylvaleric Acid	30	31.36	1.43	4.52	40	44.76	1.67	11.91	100	102.43	2.43	2.43
Hexanoic acid	37.5	42.30	1.76	12.80	50	63.43	1.06	26.86	125	130.634	1.43	4.51
**Analyte**	**L7**				**L8**							
	**C** **(µg/mL)**	**BCC** **(µg/mL)**	**RSD** **(%)**	**Accuracy** **RE (%)**	**C** **(µg/mL)**	**BCC** **(µg/mL)**	**RSD** **(%)**	**Accuracy RE (%)**				
Acetic acid	500	504.37	1.35	0.87	800	799.21	1.01	0.10				
Propionic acid	250	259.24	1.75	3.70	400	392.54	1.69	1.87				
Butyric acid	500	493.29	3.16	1.34	800		2.78					
Iso-Valeric acid	100	101.67	2.83	1.67	160	158.87	2.00	0.71				
Valeric acid	25	25.12	1.65	0.49	40	40.08	1.59	0.21				
4-Methylvaleric Acid	200	197.86	0.72	1.07	320		1.25					
Hexanoic acid	250	244.40	1.37	2.24	400		1.84					

**Table A3:** Repeatability assessment of the method using precision given as relative standard deviation RSD (%) and accuracy given as relative error (RE) (%) of individual analytes in QC samples.

	Caecum				Plasma			
	Con Calculated (µg/mL)	Con Measured (µg/mL)	Accuracy (RE%)	Precision (CV%)	Con Calculated (µg/mL)	Con Measured (µg/mL)	Accuracy (RE%)	Precision (CV%)
Acetic acid	1835.92	1853.92	0.98	9.93	107.94	97.55	9.62	15.64
Propionic acid	596.94	373.73	37.39	7.34	3.89	n.d.	N/A	N/A
Butyric acid	759.23	584.72	22.98	7.49	0.00	n.d.	N/A	N/A
Iso-Valeric acid	15.02	11.07	26.28	10.49	0.00	6.12	6.12	5.88
Valeric acid	37.41	28.68	23.33	7.87	0.21	0.17	18.45	8.65
4-Methylvaleric Acid	N/A	N/A	N/A	N/A	N/A	N/A	N/A	N/A
Hexanoic acid	1835.92	1853.92	0.98	9.93	107.94	97.55	9.62	15.64

**Table A4:** Recovery percentages obtained using acidification with phosphoric acid and succinic acid (%).

Analyte	Phosphoric Acid	Succinic Acid
Acetic acid	177.41	102.9
Propionic acid	124.69	101.08
Butyric acid	111.82	95.98
Iso-Valeric acid	124.68	110.98
Valeric acid	121.83	115.26
4-Methylvaleric Acid	114.09	105.8
Hexanoic acid	118.4	104

**Table A5:** Validation parameters of standards addition including linear regression equation, correlation coefficient of the standard addition method (R^2^), concentrations calculated by external calibration and by standard addition method.

Tissue	Analyte	Regression Linear Equation	R^2^	Concentration Calculated by External Calibration (µg/mL)	Concentration Calculated by Standard Addition Method (µg/mL)
Cecum	Acetic acid	1.5317x + 465.4086	0.9885	484.91	303.85
	Propionic acid	0.6165x + 129.5131	0.9841	128.92	210.1
	Butyric acid	0.8578x + 181.2958	0.9896	184.96	211.35
	Iso-Valeric acid	Y = 0.9646x + 3.0423	0.9855	3.51	3.53
	Valeric acid	Y = 0.9111x + 8.3260	0.9857	8.61	9.14
Plasma	Acetic acid	Y = 2.4065x + 24.3615	0.9884	28.08	10.12
Feces	Acetic acid	Y = 1.9184x + 76.8450	0.9694	87.4	40.06
	Propionic acid	Y = 1.4545 + 22.1196	0.9291	20.54	15.21
	Butyric acid	Y = 1.1192x + 23.013	0.9821	25.48	20.56
	Iso-Valeric acid	Y = 0.9171x + 1.4114	0.9724	1.50	1.54
	Valeric acid	Y = 0.8817x + 3.4704	0.9774	3.68	3.94
Liver	Acetic acid	Y = 2.6509x + 85.0558	0.9840	92.86	32.09
	Propionic acid	Y = 1.9455x − 0.2261	0.8621	0	0.12
	Valeric acid	Y = 1.3085x − 2.5070	0.9760	0.33	1.92

**Table A6:** Coefficient of variation (CV) in % for each analyte in each matrix at two concentration levels.

Analyte	Plasma		Feces		Cecum		Liver		Adipose Tissue	
	Low	High	Low	High	Low	High	Low	High	Low	High
Acetic acid	11.91	4.37	34.49	8.54	30.32	12.79	4.43	2.20	2.91	1.22
Propionic acid	2.11	1.63	9.40	4.96	9.55	3.47	1.57	3.11	1.81	2.99
Butyric acid	4.58	1.11	8.16	2.53	33.28	9.41	3.34	1.54	4.66	1.85
Iso-Valeric acid	4.84	7.09	7.09	6.51	3.71	5.66	4.99	6.69	6.06	4.66
Valeric acid	1.39	2.30	31.28	4.91	12.84	5.09	3.59	2.77	3.85	7.43
4-Methylvaleric Acid	1.09	2.24	2.87	1.69	3.94	1.77	3.55	2.78	3.12	5.75
Hexanoic acid	2.76	3.13	3.04	3.07	3.17	2.52	4.59	3.86	5.05	6.60

**Table A7:** Repeatability of individual analytes in feces and plasma given as relative standard deviation RSD (%).

Analyte	Feces	Plasma
Acetic acid	8.0	14.2
Propionic acid	4.7	25.3
Butyric acid	1.4	n.d.
Iso-Valeric acid	3.6	44.0
Valeric acid	2.0	49.5
4-Methylvaleric Acid	n.d.	n.d.
Hexanoic acid	4.2	n.d.
